# The effects of mechanical tactile stimulation on corticospinal excitability and motor function depend on pin protrusion patterns

**DOI:** 10.1038/s41598-019-53275-2

**Published:** 2019-11-13

**Authors:** Sho Kojima, Shota Miyaguchi, Ryoki Sasaki, Shota Tsuiki, Kei Saito, Yasuto Inukai, Naofumi Otsuru, Hideaki Onishi

**Affiliations:** 1 Institute for Human Movement and Medical Sciences, Niigata University of Health and Welfare, 1398 Shimami-cho, Kita-Ku, Niigata-City, Niigata 950-3198 Japan; 2 Graduate School of Health and Welfare, Niigata University of Health and Welfare, 1398 Shimami-cho, Kita-Ku, Niigata-City, Niigata 950-3198 Japan; 3 Rehabilitation Center of Shiobara Hot Spring Hospital, Tochigi Medical Association, 1333 Shiobara, Nashushiobara-City, Tochigi 329-2921 Japan

**Keywords:** Excitability, Sensorimotor processing

## Abstract

Somatosensory stimulation modulates corticospinal excitability. Mechanical tactile stimulation (MS) activates cortical activity depending on tactile stimulation patterns. In this study, we examined whether the effects of mechanical tactile stimulation on corticospinal excitability and motor function depend on different pin protrusions patterns. This single-blind study included 18 healthy subjects. Two types of MS interventions were used: repetitive global stimulus (RGS) intervention was used to stimulate the finger by using 24 pins installed on a finger pad, and sequential stepwise displacement stimulus (SSDS) intervention was used to stimulate the finger by moving a row of 6 pins between the left and right sides on the finger pad. MS interventions were applied to the right index finger for 20 min (stim on/stim off, 1 s/5 s) at a frequency of 20 Hz. After RGS intervention, motor evoked potentials (MEPs) by transcranial magnetic stimulation were observed to be significantly smaller than pre-intervention MEPs; however, motor function using the grooved pegboard task remained unchanged. After SSDS intervention, MEPs were significantly larger and motor function significantly improved compared with pre-intervention values. Our results demonstrated that MS intervention can modulate corticospinal excitability and motor function and that the effects of MS intervention depend on MS intervention patterns.

## Introduction

Prolonged and/or repetitive somatosensory stimulation, including electrical stimulation and tactile stimulation, may prove to be useful rehabilitation tools as they reportedly modulate cortical and corticospinal excitability^1–3^, motor function^4,5^ and sensory skills^6–8^ in both healthy subjects and stroke patients^9–12^. Studies of healthy subjects have reported that electrical stimulation of the median nerve increases corticospinal excitability and motor function in the fingertips^1,2,4,5^. Moreover, a study of stroke patients reported that electrical stimulation of both the median and ulnar nerves improved motor function of the upper limbs as well as the degree of muscle tone^9,10^. Accordingly, interventions using various somatosensory stimulations have been established as useful rehabilitation tools.

Repetitive sensory stimulation, including electrical or mechanical tactile stimulation (MS), is a somatosensory input tool that has been reported to alter both neurophysiological and sensory skills^6–8,13–17^. For example, MS intervention for 3 h was shown to decrease the two-point discrimination threshold, while increasing primary somatosensory cortex (S1) activity, with a corresponding correlation observed between these effects^7,8^. Moreover, previous studies have reported a projection from the S1 to the primary motor cortex (M1)^18–20^. In addition, animal studies have suggested that M1 excitability increases at rest following the cooling of S1, and decreased excitation of the pyramidal cells in S1 may alter M1 activity via long-range connections^21,22^. Based on these studies, we hypothesized that MS intervention may modulate the excitability of M1 following the modulation of S1 excitability, and we reported that repetitive global stimulus (RGS) intervention, which involves stimulating the entire finger pad at the same time, decreased corticospinal excitability, whereas sequential stepwise displacement (SSDS) intervention, which involves alternating the stimulation of the left and right sides of the finger pad, increased corticospinal excitability^23^. Furthermore, transcranial direct current stimulation (tDCS) involving noninvasive brain stimulation has indicated that stimulation on M1 can increase corticospinal excitability and motor function^24–26^. Based on these studies, we hypothesised further that RGS and/or SSDS intervention may modulate motor function while also modulating corticospinal excitability.

In spite of several studies investigating the correlation between the modulation of corticospinal excitability and motor function^26–31^, only a few studies have observed a significant correlation between the modulation of corticospinal excitability and motor function using transcranial magnetic stimulation (TMS). Therefore, it is possible that the level of motor evoked potential (MEP), indicating corticospinal excitability, induced by single-intensity TMS is insufficient to capture the modulation of motor function. Thus, in the current study, we developed a more sensitive measure for recording corticospinal excitability using input–output curves (I-O curves), plotting the MEP amplitudes measured by a range of stimulus intensities. Indeed, many studies have demonstrated that I-O curves are modulated by somatosensory input interventions^28,30,32–38^. I-O curves can measure the modulation of pyramidal cell excitability with different thresholds in greater detail compared with other methods, including by comparing changes in MEPs due to inhibitory or facilitatory mechanisms by paired-pulse TMS or by relying on changes in MEPs at a single-pulse TMS^39–41^. Moreover, I-O curves believed to change with the distribution of excitability in the corticospinal system or in the spatial distribution of excitable elements in the cortex^42^. Therefore, the use of I-O curves in the current study allowed for more detailed measurements of the modulation of corticospinal excitability.

Collectively, the aim of this study was to investigate whether the effects of mechanical tactile stimulation on corticospinal excitability and motor function depend with pin protrusions pattern and to evaluate the correlation between both modulations by using I-O curves and motor function tasks.

## Results

**Figure 1:**
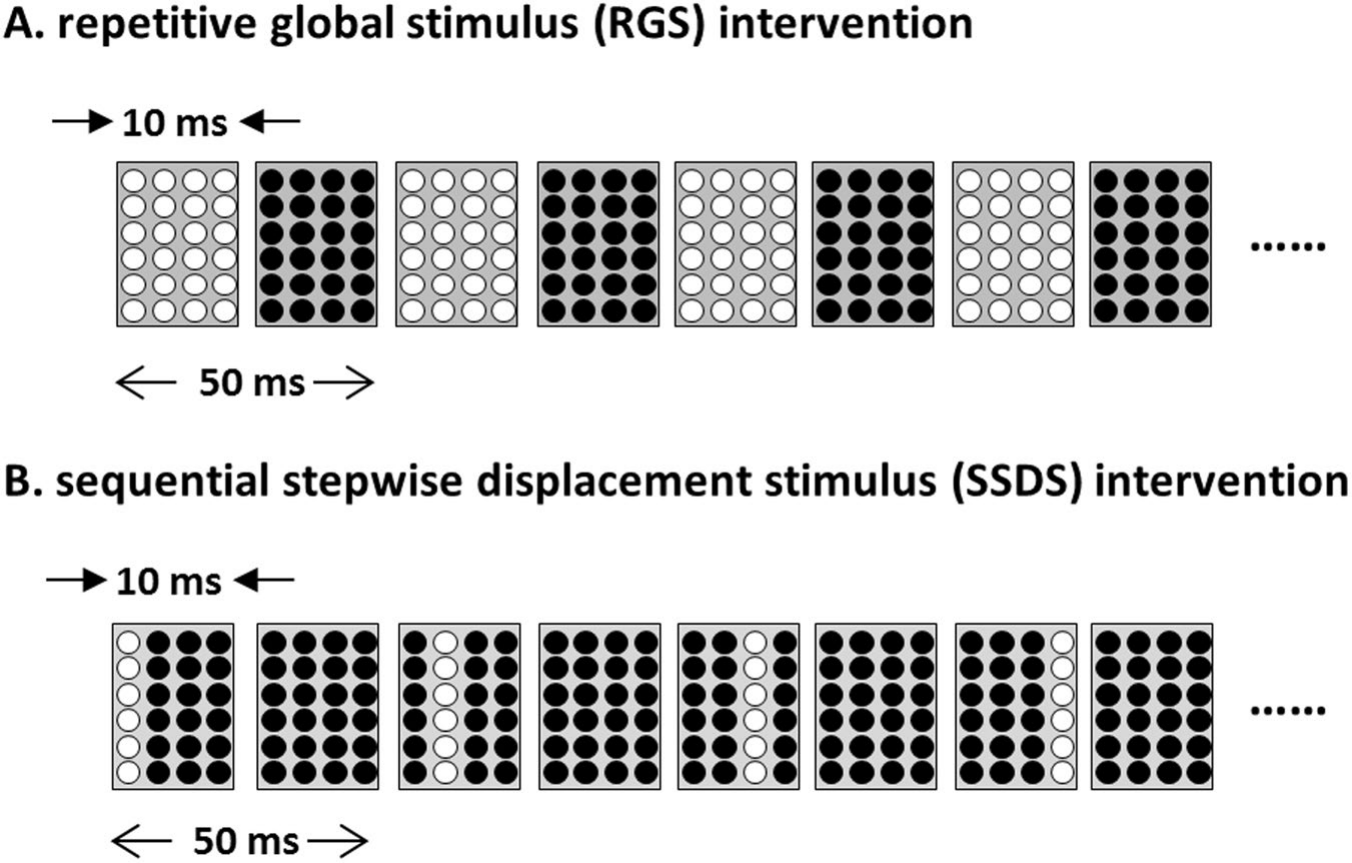
The MS intervention patterns. Figure shows the patterns of mechanical tactile intervention. RGS intervention stimulated the index finger with 24 pins installed in the finger pad (**A**). SSDS intervention stimulated the finger by moving the row of six pins between the left and right sides on the finger pad (**B**).

Figure 1 shows the intervention patterns. The mean resting motor threshold (RMT) intensity [mean ± standard division (SD)] was 51.7 ± 7.9% maximal stimulator output (% MSO) for the RGS intervention and 52.8 ± 8.2% MSO for the SSDS intervention. Paired t-tests indicated that the differences in the % MSO between interventions were not statistically significant.

### Effects of MS intervention on corticospinal excitability

Table 1 presents the mean MEP amplitudes evoked by each TMS intensity pre- and post-intervention. Three-way repeated measure analysis of variance (ANOVA) revealed a significant main effect of INTENSITY (*F*_*(5*, *85)*_* = 70*.*565*, *P = 8*.*8*2*93E-*2*9*, *partial eta squared (η*^2^*) = 0*.*806*) and a significant three-way interaction of INTERVENTION × INTENSITY × TIME (*F*_*(5*, *85)*_* = 9*.*730*, *P = *2.2*493E-7*, *partial η*^2^* = 0*.*364*). The effect of INTERVENTION (*F*_*(1*, *17)*_* = *2.*138*, *P = 0*.*16*2, *partial η*^2^* = 0*.*11*2) and TIME (*F*_*(1*, *17)*_* = 0*.*582*, *P = 0*.*082*, *partial η*^*2*^* = 0*.*167*) did not reach the level of statistical significance.

**Table 1:** MEP amplitude, I-O curve slope and performance time of pre- and post-intervention (mean ± standard error of mean).

*MEP amplitude (mV)*	*Stimulus intensity*	*RGS intervention*			*SSDS intervention*		
		*PRE-INTERVENTION*	*POST-INTERVENTION*	*P-value*	*PRE-INTERVENTION*	*POST-INTERVENTION*	*P-value*
	*100%RMT*	0.07 ± 0.01	0.06 ± 0.02	0.689	0.08 ± 0.02	0.08 ± 0.02	0.535
	*110%RMT*	0.28 ± 0.04	0.20 ± 0.04	0.005*	0.27 ± 0.06	0.38 ± 0.10	0.054
	*120%RMT*	0.69 ± 0.10	0.58 ± 0.11	0.072	0.80 ± 0.13	1.02 ± 0.20	0.033
	*130%RMT*	1.27 ± 0.13	1.07 ± 0.16	0.035	1.34 ± 0.21	1.59 ± 0.24	0.009
	*140%RMT*	1.79 ± 0.18	1.58 ± 0.19	0.012	1.78 ± 0.25	2.28 ± 0.33	0.00057**
	*150%RMT*	2.23 ± 0.22	2.14 ± 0.25	0.308	2.11 ± 0.29	2.59 ± 0.35	0.00078**
P-Value: t-test with Bonferroni correction (statistical significant; P < 0.008*, P < 0.0016**)							
***I-O curve***	*slope*	0.46 ± 0.05	0.43 ± 0.05	0.158	0.43 ± 0.06	0.54 ± 0.07	0.00023**
P-Value: paired t-test (statistical significant; P < 0.01**)							
***motor function***	*performance time(s)*	54.67 ± 1.48	54.46 ± 1.50	0.736	56.75 ± 1.20	53.67 ± 1.42	0.005**
P-Value: paired t-test (statistical significant; P < 0.01**)							

RGS: repetitive global sltimulus, SSDS: sequential stepwise displacement stimulus, degree of freedom; P < 0.05.

**Figure 2:**
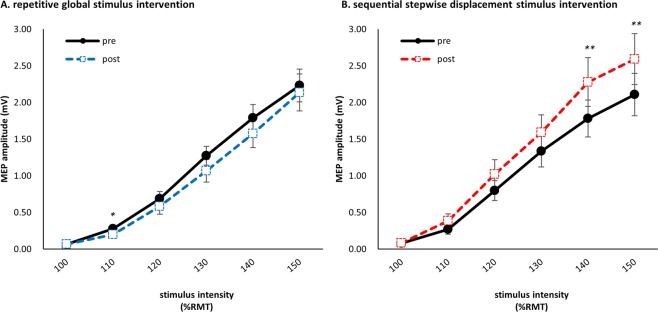
Pre- to post-intervention changes in the I-O curve plotting the MEP amplitude (mean ± standard error of mean) (*P < *0*.*008*, **P < *0*.*0016*). (**A**) In the RGS intervention, post hoc analysis showed a significant decrease in the MEP amplitude evoked by 110% RMT PRE compared with POST (*P* = *0*.*005*). (**B**) In the SSDS intervention, post hoc analysis showed a significant increase in the MEP amplitude evoked by 140, 150% RMT PRE compared with POST (140%RMT; *P = 0*.*00057*, 150%RMT; *P = 0*.*00078*).

In the RGS intervention, post hoc analysis using paired *t*-tests with Bonferroni correction showed a significant pre- to post-intervention decrease in the MEP amplitude evoked by 110%RMT (*110%RMT; P = 0*.*005*) (Fig. 2A). In the SSDS intervention, there was a significant pre- to post-intervention increase in the MEP amplitude evoked by 140, 150%RMT (*140%RMT; P = 0*.*00057*, *150%RMT; P = 0*.*00078*) (Fig. 2B). There were no significant differences in MEP amplitudes at other intensities for either MS intervention (*P > 0*.*05*).

Table 1 presents the pre- and post-intervention I-O curve slopes. Two-way repeated measure ANOVA revealed a significant main effect of TIME (*F*_*(1*, *17)*_* = 11*.*626*, *P = 0*.*003*, *partial η*^*2*^* = 0*.*406*), a significant interaction effect of INTERVENTION × TIME (*F*_*(1*, *17)*_* = 11*.*868*, *P = 0*.*003*, *partial η*^*2*^* = 0*.*411*) and nonsignificant effect of INTERVENTION (*F*_*(1*, *17)*_* = 0*.*064*, *P = 0*.*803*, *partial η*^*2*^* = 0*.*004*). Post hoc analysis showed that the slope of the post-intervention curve was significantly larger than that of the pre-intervention curve for the SSDS intervention (*P = 0*.*00023*). The pre- to post-intervention change in the slope of the curve for the RGS intervention did not reach the level of statistical significance (*P = 0*.*158*).

### Effects of MS intervention on motor function

**Figure 3:**
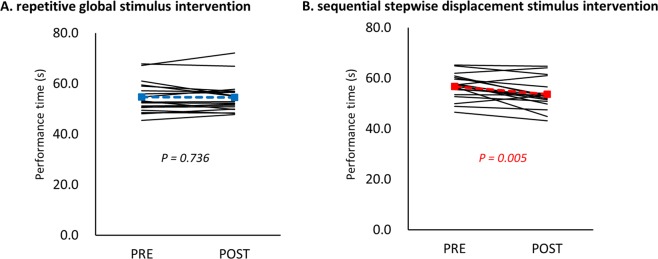
Pre- to post-intervention changes of individual subjects in motor performance of GPT. RGS intervention did not change the performance time PRE to POST, whereas the SSDS intervention decreased the performance time.

Table 1 presents the mean performance times of the pre- and post-interventions. For the performance time, two-way repeated measures ANOVA revealed a significant main effect of TIME (*F*_*(1*, *17)*_* = 9*.*013*, *P = 0*.*008*, *partial η*^*2*^* = 0*.*346*) and a significant interaction of INTERVENTION × TIME (*F*_*(1*, *17)*_* = 5*.*759*, *P = 0*.*028*, *partial η*^*2*^* = 0*.*253*) but not a significant effect of INTERVENTION (*F*_*(1*, *17)*_* = 0*.*270*, *P = 0*.*610*, *partial η*^*2*^* = 0*.*016*). For the SSDS intervention, post hoc analysis showed that the performance time of the post-intervention was faster than that of the pre-intervention (*P = 0*.*005*). However, for the RGS intervention, the pre- and post-intervention differences in performance times were not statistically significant (*P = 0*.*736*) (Fig. 3).

### Correlation between corticospinal excitability and motor function

Correlations between the differences in neurophysiological data (MEP amplitude by each TMS intensity and I-O curve slopes) and performance times were not statistically significant (*P > 0*.*05*).

## Discussion

In this study, RGS interventions were shown to decrease corticospinal excitability, whereas SSDS interventions increased corticospinal excitability. These results strongly suggest that modulation of corticospinal excitability is dependent on pin protrusion patterns of MS intervention. These findings are consistent with our previous study demonstrating that the application of an RGS or SSDS intervention (previous study: simple or lateral complex) for 20 min modulates corticospinal excitability^23^. Another previous study using identical MS reported that cortical activity depends on the pattern of MS and demonstrated further that S1 was activated by both RGS and SSDS, whereas M1 was only activated by SSDS^43^. It has also been reported that activities of the secondary somatosensory, premotor and posterior parietal cortices (PPCs) are induced by SSDS, but not RGS^44,45^. The connectivity between these areas and M1 has been shown in animal studies^46,47^, and inhibitory/facilitatory connectivity has been demonstrated between the premotor area and M1 in human studies using TMS^48,49^. Therefore, we hypothesized that activity was induced in different cortical sites by differing MS interventions. Furthermore, we investigated the modulation of MEP amplitude by using TMS of varying intensities and found that RGS intervention decreased MEP amplitudes evoked by low-intensity TMS (110%RMT), whereas SSDS intervention increased MEP amplitudes evoked by high-intensity TMS (140 and 150%RMT). Similarly, a previous study observed a prolonged silent period, described as a period of EMG silence until activity returned to pre-stimulus baseline levels, earlier than the MEP amplitudes appeared when the TMS intensity increased gradually during slight muscle contraction^50^. Low-intensity TMS was believed to evoke inhibitory interneuron activity because this silent period was associated with activity of the inhibitory interneurons and because the threshold of the inhibitory interneurons was lower than that of the facilitatory neurones^50^. Therefore, it is thought that the MEP amplitude evoked by varying TMS intensities is indicative of the interneuron excitability of different thresholds. In the current study, the effects of RGS intervention were observed for low TMS intensities, thus, indicating the excitability of the inhibitory interneurons. However, the effects of the RGS intervention were not observed for high TMS intensities, indicating the excitability of the facilitatory interneurons. In contrast, the effects of SSDS intervention were clearly observed for high TMS intensities, but not for low TMS intensities. Therefore, we postulated that the effect of MS intervention on different interneurons depends on the pattern of MS intervention. Moreover, previous study has shown that the intervention of somatosensory stimulation modulates corticospinal excitability, and the effects include intracortical inhibition, intracortical facilitation, and the firing rate of M1 neurons^32,35,51,52^. Therefore, in this study, the modulation of MS intervention may have been induced by the modulation of either spinal or intracortical excitability, and we plan to study the relationship between the effects of MS intervention and the modulation of these excitabilities in the future.

In this study, RGS intervention did not modulate motor function, whereas SSDS intervention increased motor function. Therefore, modulation of motor function appears to be dependent on the pattern of MS intervention. Previous studies have reported that somatosensory input or noninvasive brain stimulation intervention increases the performance on the peg task^4,26^. An additional study involving high-frequency electrical stimulation of the thumb and index finger also reported a decreased performance time for the peg task after intervention for 30 min^4^. Furthermore, a study using tDCS, which is a noninvasive brain stimulation method that modulates corticospinal excitability, reported that anodal tDCS on M1 decreased performance time on the pegboard, with increasing MEP amplitude^26^. Taken together, these studies indicate improved motor function following increased corticospinal excitability. Accordingly, in the current study, we demonstrated that the application of SSDS intervention for 20 min increased motor function after an increase in corticospinal excitability. Furthermore, previous studies have reported that the pattern of SSDS intervention involving the movement of stimulation in the stimulus area evoked the activity of several cortical areas related to motor movement, including the premotor cortex and PPC^44,45^. Thus, an SSDS intervention, but not an RGS intervention, can evoke activity in a motor related area, such that the motor function increases with increasing corticospinal excitability in motor related areas.

The correlations between differences in neurophysiological data (MEP amplitude by each TMS intensity and I-O curve slopes) and performance times were not statistically significant. Previous research has evaluated the correlation between corticospinal excitability and motor function pre- and post-intervention^26–29^. Cirillo *et al*.^28^ reported that continued motor training increased MEP amplitude and motor function but the authors did not observe a significant correlation. Similarly, a study using ballistic movement training reported that training increased MEP amplitudes and motor performance but also did not find a significant correlation^27^. Moreover, Kidgell *et al*.^26^ reported that anodal tDCS on M1 increased MEP amplitudes and motor performance on the pegboard task and decreased short-interval intracortical inhibition; however, they did not observe a significant correlation between these indications. This lack of significant correlations is likely due to the use of modulation of MEP amplitudes evoked by single-intensity TMS^27^ or the I-O curve slope^27,28^ in their analyses. On the other hand, Muellbacher, *et al*.^53^ reported that a repetitive pinch movement for 60 min increased MEP amplitudes with increasing pinch force, and that a significant positive correlation existed between these indications. Garry, *et al*.^31^ also reported a correlation between the facilitation of MEP amplitude and motor function after simple motor training. McDonnell and Ridding^54^, who recorded MEP by TMS, found MEP facilitation using TMS intensity that evoked MEPs of 0.5–1 mV after the repetitive grooved pegboard task (GPT); however, they did not observe a significant correlation between MEP facilitation and GPT modulation. Furthermore, the authors suggested there may be a complex modulation in motor cortical activation with a more complex GPT. Accordingly, we investigated the modulation of corticospinal excitability by using I-O curves, reflecting corticospinal excitability in more detail by recording corticospinal excitability at various TMS intensities. However, we observed no correlations between the differences in neurophysiological data (MEP amplitude by each TMS intensity and I-O curve slopes) and the performance times of the peg task. In previous studies investigating the association between motor function and MEP modulation, significant correlations were reported between MEPs evoked by single-intensity TMS (120%RMT) and motor performance of a simple task (pinch and simple peg task)^31,53^, but not between MEPs and complex motor tasks^31,54^. Moreover, Williams *et al*.^29^ reported that complex motor performances of the non-dominant hand were correlated with transcallosal inhibition, indicating inhibition from the dominant to the non-dominant hemisphere. Based on these studies, corticospinal excitability of MEPs may not reflect modulation of complex motor function such as GPT. However, the details need to be clarified through meta-analysis of either the r-values or other statistics reported in previous studies. Therefore, we plan to conduct a meta-analysis to examine the correlation between these neurophysiological makers (intracortical inhibition/facilitation) and complex motor function.

In summary, we investigated the effects of MS intervention on corticospinal excitability and motor function. We found that RGS intervention decreased corticospinal excitability, but did not change motor function, whereas SSDS intervention increased both corticospinal excitability and motor function. Collectively, our novel results show that MS interventions can modulate corticospinal excitability and motor function and that the effects of the intervention are dependent on the pattern of MS.

## Methods

### Participants

Eighteen healthy volunteers (age range, 20–27 years; mean ± SD, 22.0 ± 2.2 years) participated in this study. None of the participants reported taking drugs or medications known to affect the central nervous system. This study was approved by the Ethics Committee of Niigata University of Health and Welfare and was conducted in accordance with the Declaration of Helsinki. All participants provided written informed consent before participating in this research.

### Corticospinal excitability measurement

This study used the I-O curve to indicate corticospinal excitability by measuring MEP amplitudes. MEPs were recorded from the right first dorsal interosseous (FDI) muscle by using silver/sliver chloride electrodes in a belly-tendon montage. Electromyogram signals were amplified by 100X (A-DL-720-140 Amplifier; 4 Assist, Tokyo, Japan), digitized at 10 kHz by using an A/D converter (Power Lab 8/30; AD instruments, Colorado Springs, CO, USA) and analysed using Lab Chart 7 (AD instrument). We used monophasic-pulse TMS to elicit MEP, with the TMS delivered by a figure-eight-shaped coil (95 mm diameter) connected to a Magstim 200 square (Magstim, Dyfed, UK). The coil was held with the handle pointing backwards and laterally at an angle, approximately 45° to the sagittal plane. The optimal spot for eliciting MEPs was defined as the point where the TMS consistently evoked a large MEP from the right FDI and was carefully determined for each participant. The optimal coil position was marked on a cap worn by the participants. The position and orientation of the coil was monitored throughout the experiment by magnetic resonance imaging (MRI) using a Visor2 TMS Neuronavigation System (Emagine Medical Imaging Solutions GmbH, Berlin, Germany). The optimal spot on the FDI muscle was recorded, and the coil was manually held in place to maintain the position during the experiment. In addition, the TMS was performed over the hotspot of the FDI when the position of the coil was within 1 mm of the hotspot and the rotation was within 2 degrees. Before the experiment, T1-weighted MRI was performed using a 1.5 Tesla scanner (Signa HD, GE Healthcare, Milwaukee, WI, USA). The RMT was defined as the lowest stimulus intensity (% MSO) that induced a MEP in the relaxed FDI muscle with 50μV peak-to-peak amplitude in five out of 10 consecutive stimulations. The I-O curves were then randomly measured by TMS at six intensity levels, ranging from 100% to 150% RMT, in steps of 10% RMT. Twelve stimuli were recorded at each intensity level, with an interstimulus interval of 5–6 s.

### Motor function measurement

The GPT was used to assess motor function in this study^54,55^. Briefly, the pegboard test composed of 25 holes with randomly positioned slots and pegs, with a key on one side. During the test, the pegs (3 mm in diameter) must be rotated to match the hole before they can be inserted. The GPT is more difficult than the conventional pegboard test. For the test, subjects were encouraged to pick up the pegs from a well and place all the 25 pegs as quickly as possible in a vertical array of holes using only the index finger, middle finger and thumb. Motor function was quantified by recording the time it took to complete the task (performance time) using a stopwatch. Each participant was tested two times, and the average score was used as a measure of motor function.

### Mechanical tactile stimulation intervention

**Figure 4:**
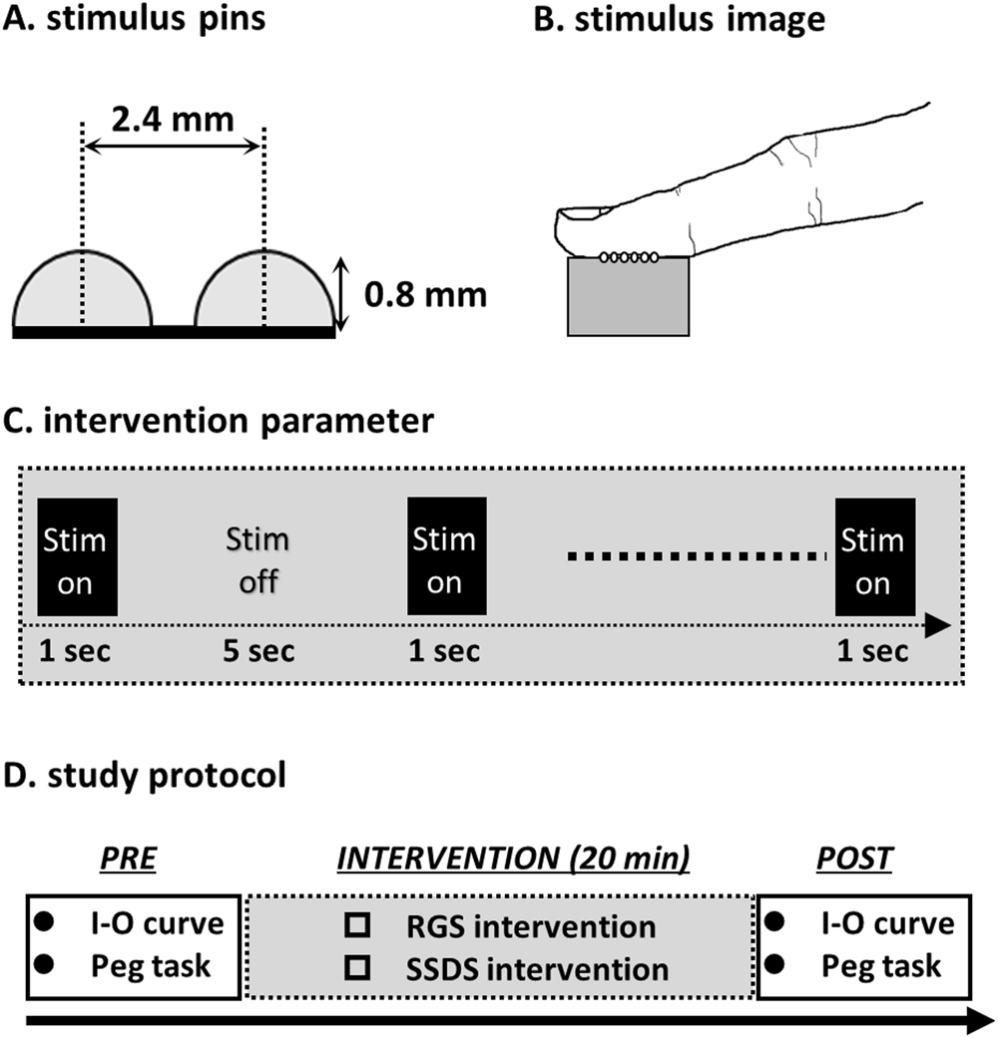
MS and study protocol. (**A**) Figure shows the mechanical tactile stimulator. Measurements for each pin are as follows: 1.3 mm diameter and 0.8 mm protrusion height. (**B**) The mechanical tactile stimulator comprised 24 tiny plastic pins applied to the tip of the right index finger. (**C**) MS intervention was applied for 20 min (stim on/stim off, 1 s/5 s) at a frequency of 20 Hz. (**D**) I-O curve and motor function were measured prior to intervention (PRE). Each MS intervention (either RGS or SSDS) was applied for 20 min. After MS intervention (POST), the I-O curve and motor function were measured again.

The mechanical tactile stimulator consisted of 24 tiny plastic pins driven by piezoelectric actuators (TI-1101; KGS, Saitama, Japan), which are illustrated in Fig. 4A. The pins had a diameter of 1.3 mm, and the height of the protrusion was 0.8 mm, with a pushing force of 0.031–0.12 N/pin^23,56,57^. The distance between pins was set at 2.4 mm. The protruding MS was applied to the tip of the right index finger for 10 ms, as shown in Fig. 4B. Figure 1 shows the patterns of tactile intervention. Two MS interventions were used in the current study. The RGS intervention stimulated the index finger with 24 pins installed in the finger pad (Fig. 1A), whereas the SSDS intervention stimulated the index finger by moving the row with six pins between the left and right sides of the finger pad (Fig. 1B). The differences between these interventions represented the stimulus stepwise displacement. MS interventions were applied for 20 min (stim on/stim off, 1 s/5 s) at a frequency of 20 Hz (Fig. 4C). During the MS intervention, participants were instructed to maintain a relaxed hand position and to look at a target, thus, directing their attention away from the right hand. The FDI was monitored for a lack of contraction during the MS intervention.

### Study design

This study used a repeated measures design to assess corticospinal excitability and motor function pre- and post-MS intervention (Fig. 4D). For the experiment, participants were seated comfortably in a chair with their forearm pronated. Before the intervention (PRE), baseline I-O curves and motor function were measured. Thereafter, one of the MS interventions (either RGS or SSDS) was performed for 20 min. After the MS intervention (POST), I-O curves and motor function were measured again. The interventions were performed in a random order, with an interval of at least one week between conditions.

### Data and statistical analysis

Mean MEP amplitudes were calculated from the peak-to-peak amplitudes of the recorded MEP for each TMS intensity and were plotted against the stimulus intensity^58,59^. The slope of the I-O curve was calculated on the basis of a generalized linear model. Motor function, which was based on performance time, was calculated as the mean of the two trials.

SPSS software version 24 (IBM SPSS, Armonk, New York, USA) was used to perform statistical analyses. MEP amplitudes were analysed by three-way repeated measures ANOVA [INTERVENTION (RGS, SSDS) × INTENSITY (100, 110, 120, 130, 140 and 150%RMT) × TIME (PRE, POST)]. The effect size of the ANOVA was calculated as partial η^2^. Post hoc analyses were performed using paired *t*-tests with Bonferroni correction to compare pre and post values. The statistical significance of MEP amplitudes was set at P < 0.008 (0.05/n, n = 6; a number of TMS intensity). The mean I-O curve slope and motor function (performance time) were analysed by two-way repeated measures ANOVA (INTERVENTION × TIME) with the effect size calculated using partial η^2^. Post hoc analyses were performed using paired *t*-tests to compare pre and post values. Correlations between the modulation of neurophysiological data (MEP amplitude by each TMS intensity and I-O curve slopes) and that of performance time (% performance time) were assessed using the differences (POST – PRE) calculated using Pearson’s correlation coefficient or Spearman’s rank correlation coefficient for parametric and nonparametric data, respectively. Statistical significance was set at P < 0.05.

## Data Availability

The datasets generated during and/or analysed during the current study are available from the corresponding author.
